# In situ atomic-resolution imaging of water vapor–driven multistep oxidation dynamics in strontium cobaltite

**DOI:** 10.1126/sciadv.adx8890

**Published:** 2025-08-22

**Authors:** Zhenzhong Yang, Ke Qu, Yifeng Zhao, Le Wang, Libor Kovarik, Peter V. Sushko, Yingjie Lyu, Jianbing Zhang, Pu Yu, Chungang Duan, Yingge Du

**Affiliations:** ^1^ Key Laboratory of Polar Materials and Devices, Department of Electronics, East China Normal University, Shanghai 200241, China.; ^2^ Physical and Computational Sciences Directorate, Pacific Northwest National Laboratory, Richland, WA 99354, USA.; ^3^ State Key Laboratory of Low Dimensional Quantum Physics and Department of Physics, Tsinghua University, Beijing, China.; ^4^ Collaborative Innovation Center of Extreme Optics, Shanxi University, Shanxi 030006, China.

## Abstract

Understanding how water vapor interacts with transition metal oxides (TMOs) is critical for tailoring material properties to improve performance and enable new technologies. Despite extensive research efforts, atomic-scale mechanisms underpinning dynamic reactions and reaction-induced phase transitions remain elusive. Here, we use in situ environmental transmission electron microscopy to investigate how water vapor oxidizes vacancy-ordered SrCoO_2.5_ at moderately elevated temperatures, demonstrating that water molecules can initiate oxidation more effectively than oxygen under comparable conditions. We discover a distinct “staging” behavior during the oxidation process: A fully ordered intermediate phase, SrCoO_2.75_, forms before transitioning into a near-perovskite SrCoO_3−δ_. In addition, antiphase boundaries, originating at step terraces of SrTiO_3_, alleviate strain by creating reversible nanoscale “gaps” during lattice contraction under oxidation, providing a pathway for preserving structural integrity throughout redox cycling. This work provides atomic-level guidance for engineering TMOs by leveraging water vapor to control their redox behavior and tailor functional properties.

## Teaser

The multistep oxidation driven by water vapor is directly observed at the atomic scale using in situ ETEM.

## INTRODUCTION

The physical and chemical properties of functional oxides, including electrical conductivity, oxygen diffusivity, and electrocatalytic reactivity, are highly sensitive to variations in oxygen stoichiometry, often mediated by topotactic phase transitions (TPTs) (*1*–*5*). These TPTs, driven by the intercalation and extraction of oxygen ions, underpin many of these key functionalities in complex oxides, like strontium cobaltites and strontium ferrites. Therefore, atomic-scale visualization of these dynamic processes is essential for elucidating the mechanisms underpinning these transitions and for the rational design of advanced materials and devices, including water oxidation catalysts, oxygen permeation membranes, and solid oxide fuel cells (*6*–*9*).

Recent research demonstrates that both isolated oxygen vacancies (V_O_) and highly ordered oxygen vacancy channels (OVCs) can strongly influence catalytic reactions and oxygen transport (*10*–*13*). When water vapor is involved, hydroxyl species (OH^−^) or protons (H^+^) may diffuse more readily than oxygen anions (O^2−^), accelerating redox reactions and potentially stabilizing or destabilizing intermediate oxide phases (*14*). Despite growing interest in these phenomena, the fundamental details of how water vapor drives structural and electronic transformations in vacancy-ordered oxides remain insufficiently understood. Thus, real-time observation of the interaction between water vapor and transition metal oxides (TMOs) is vital to understanding the associated microstructural dynamics.

Strontium cobaltite (SrCoO*_x_*, 2.5 ≤ *x* ≤ 3.0) is an ideal system for investigating such water-induced oxidation, as its crystal structure and physical properties undergo marked changes in response to variations in oxygen content. For example, SrCoO_2.5_ is an antiferromagnetic insulator that exhibits a brownmillerite (BM-SCO) structure with alternating layers of octahedral CoO_6_ and tetrahedral CoO_4_ forming two-dimensional OVCs. By contrast, SrCoO_3_ has a perovskite (P-SCO) structure with ferromagnetic metallic properties (*15*). While the reversible transition between BM-SCO and P-SCO has been widely studied (*13*, *16*), direct atomic-scale observation of the microstructural evolution during this redox process, particularly as a result of water exposure, remains lacking. This is largely due to the challenges associated with the oxidation of TMOs under controlled environments, which hinder real-time, atomic-scale imaging. Recent advances in environmental transmission electron microscopy (ETEM), as well as in situ gas flow holders, have enabled researchers to directly capture real-time, atomic-level microstructural evolutions, providing insights into key processes such as oxidation (*17*–*19*), catalysis (*20*–*22*), and moisture-induced crystal interactions (*23*).

In this work, we use in situ ETEM to reveal how controlled water vapor can initiate a reversible TPT between BM-SCO and perovskite-like SrCoO_3−δ_ (PL-SCO). Our findings reveal that water vapor, even at very low partial pressure (~10^−2^ mTorr), is more effective in promoting the oxidation of BM-SCO compared to molecular oxygen. We show that water vapor effectively promotes oxidation through a two-step process, forming a fully ordered intermediate SrCoO_2.75_ phase before transitioning into a near-perovskite SrCoO_3−δ_. Moreover, we demonstrate the critical role of antiphase boundaries (APBs), which form at substrate step terraces, in relaxing strain via nanoscale “gaps”. These reversible gaps could mitigate structural deterioration across the redox reaction (*24*, *25*). By coupling experimental results with density functional theory (DFT) calculations, we provide an atomic-level blueprint for leveraging water vapor to tune phase behavior and defect structures in vacancy-ordered oxides.

## RESULTS

### In situ observation of structural changes induced by water vapor oxidation

**Fig. 1.**
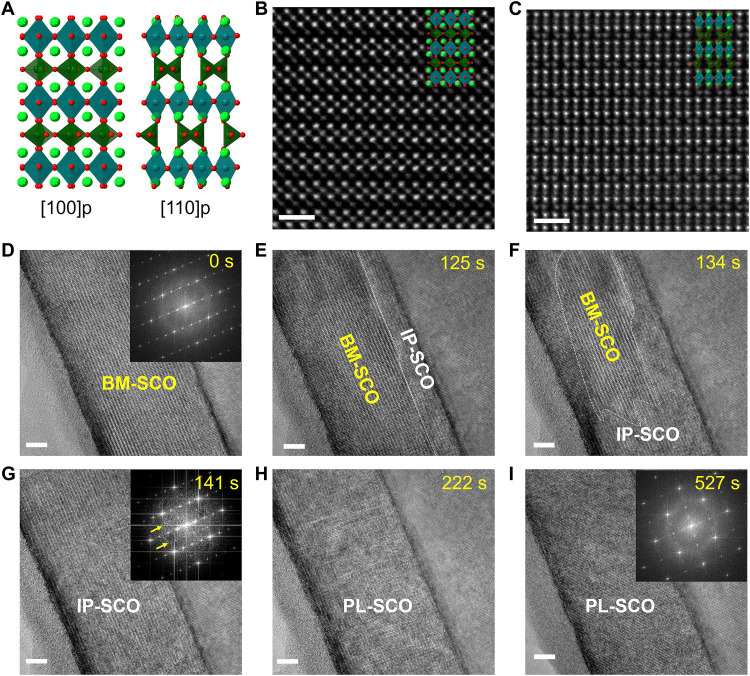
Water vapor–induced multistep oxidation in BM-SCO. (**A**) Atomic model of BM-SCO, highlighting alternating layers of CoO_6_ octahedra (dark cyan) and CoO_4_ tetrahedra (green). (**B** and **C**) Representative HAADF-STEM images of BM-SCO viewed along the [100] and [110] zone axis, respectively. Scale bars, 1 nm. (**D** to **I**) Time-series HR-TEM snapshots captured during in situ TEM, along with corresponding FFT patterns (insets), showing the phase transition from BM-SCO to IP-SCO and eventually PL-SCO at ~0.01 mTorr water vapor and 100°C. Scale bars, 5 nm.

BM-SCO thin films were grown on (001) oriented 0.7 or 0.5 wt % Nb-doped STO substrates using pulsed laser deposition (*16*). As illustrated in Fig. 1A, BM-SCO exhibits an alternating stacking of CoO_6_ octahedra (dark cyan) and CoO_4_ tetrahedra (green). OVCs formed along the CoO_4_ layers (*26*, *27*) evidenced by the large Sr-Sr spacing of ~4.36 Å across these tetrahedral layers, compared to ~3.52 Å in the octahedral sublayers (*28*). Figure 1 (B and C) displays high-angle annular dark-field scanning transmission electron microscopy (HAADF-STEM) images of this vacancy-ordered structure. Because the contrast in annular bright field (ABF)–STEM images is sensitive to atomic number (with a ~*Z*^1/3^ dependence, where *Z* represents the atomic number), it is particularly effective for simultaneously visualizing both heavier and lighter atoms (*29*, *30*), enabling us to observe Sr and Co as well as oxygen atoms in this study. Therefore, the ABF-STEM image, along with the line intensity profile displayed in fig. S1, provides a detailed view of the OVC structure in BM-SCO.

To investigate water vapor–driven oxidation, we used ETEM to promote the interaction between water and cross-sectional BM-SCO thin-film samples at a mild temperature and at ~0.01 mTorr water vapor. In situ ETEM experiments were performed at various temperatures. While rapid corrosion and the formation of an amorphous phase (fig. S2) were observed at room temperature, a multistep oxidation pathway emerged at elevated temperatures, evident in the real-time high-resolution TEM (HR-TEM) images (movie S1). Figure 1 (D to I) shows that BM-SCO gradually oxidizes to a perovskite-like SrCoO_3−δ_ (PL-SCO) through an ordered intermediate phase (IP-SCO). Notably, the IP-SCO diffraction pattern reveals additional superstructure diffraction spots (Fig. 1G and fig. S3) that are not present in pristine BM-SCO (Fig. 1D). The ordered IP-SCO then transformed into PL-SCO during continued oxidation. Figure 1H reveals the gradual emergence of a vertically ordered structure, which progressively diminishes in clarity of the ordering within the IP-SCO, suggesting a decrease in oxygen vacancy ordering. This is further confirmed by the HAADF-STEM image in fig. S4, where the oxygen vacancy distribution appears less ordered, exhibiting both vertical and horizontal arrangements.

### Atomic-scale structure evolution during water vapor oxidation

**Fig. 2.**
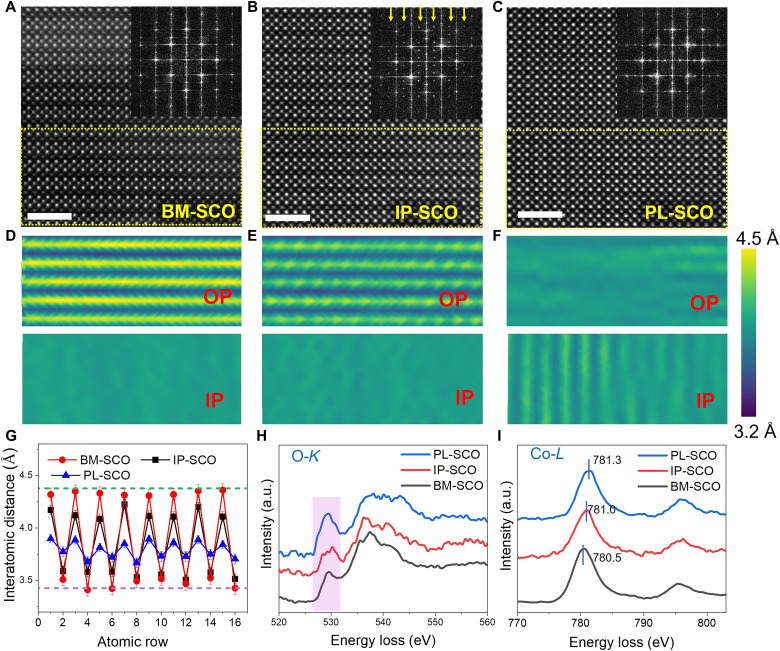
Atomic-scale structure evolution of SCO during water vapor oxidation. (**A** to **C**) HAADF-STEM images of BM-SCO, IP-SCO, and PL-SCO, respectively, with corresponding FFT patterns (insets). Scale bars, 2 nm. (**D** to **F**) Lattice parameter measurements from the yellow dashed boxes in (A) to (C), showing progressive changes in out-of-plane and in-plane lattice distances. (**G**) Out-of-plane Sr-Sr interatomic distance maps revealing the reduction in layer spacing across the vacancy-containing sublayers as oxidation proceeds. (**H** and **I**) EELS spectra of the O-*K* and Co-*L* edges, demonstrating increased prepeak intensity of O-*K* (pink shaded) and positive shift in Co valence states as oxidation proceeds.

To elucidate the structural details, aberration-corrected HAADF-STEM images of BM-SCO, IP-SCO, and PL-SCO were obtained (Fig. 2, A to C). The out-of-plane and in-plane lattice parameter maps in Fig. 2 (D to F) illustrates a progressive reduction in the Sr-Sr distance across the tetrahedral sublayers from BM-SCO to IP-SCO and lastly to PL-SCO. As oxidation proceeds, the Sr-Sr distance across the OVC channels shrinks from ~4.3 Å in BM-SCO to ~4.1 Å for IP-SCO, then to ~3.8 Å for PL-SCO as shown in Fig. 2G. In IP-SCO, using the out-of-plane lattice spacing as an indicator of oxygen content (*31*, *32*) (fig. S5), the composition of the oxygen-deficient layers was estimated as CoO_1.44±0.07_, yielding an overall stoichiometry of SrCoO_2.72±0.035_ for this half-oxidized phase. Considering the chemistry of the strontium cobaltite, this intermediate phase can be described as part of the homologous series SrCoO_(3*n*−1)/*n*_ (*n* = 2 to ∞), with the half-oxidized IP-SCO phase corresponding to SrCoO_2.75_ (*n* = 4). The HAADF-STEM image in Fig. 2B further confirms that the IP-SCO exhibits an ordered structure, similar to BM-SCO. Moreover, additional diffraction spots emerge in the fast Fourier transformation (FFT) pattern of IP-SCO, consistent with the observations from HR-TEM (Fig. 1G). We note that such an ordered intermediate state was also observed in SrFeO_2.75_ as a result of an intercalation-type oxygen diffusion in BM-SrFeO_2.5_ (*4*).

These observations are consistent with DFT-calculated structural models (fig. S6), which predict that the CoO_4_ tetrahedra in BM-SCO transition to CoO_5_ pyramids in IP-SCO. The computed Sr-Sr distances in the CoO_5_ and CoO_6_ layers are ~4.0 and ~3.6 Å, respectively, matching the experimental measurements (Fig. 2G). In the PL-SCO phase, the HAADF-STEM image (Fig. 2C) suggests that the structure adopts a perovskite-like arrangement with no obvious vacancy ordering. However, the weak modulation in Sr-Sr interatomic distances observed in both in-plane and out-of-plane (Fig. 2, C, F, and G) suggests the presence of a small amount of V_O_.

The evolution of Co valence states across the three phases was investigated using electron energy-loss spectroscopy (EELS), focusing on the O *K*-edge and Co *L*-edge, as shown in Fig. 2 (H and I). The O-*K* edge spectra show a clear broadening and increase in the prepeak in both IP-SCO and PL-SCO compared with BM-SCO, resulting from enhanced hybridization between O 2p and Co 3d orbitals (*16*, *33*). As shown in Fig. 2I, the Co-*L* edge spectra also demonstrate a shift to higher energy from BM-SCO to IP-SCO and further to PL-SCO, consistent with an increase in the valence of Co (*33*). The chemical shift at the Co *L*_2_ edge between BM-SCO and IP-SCO is ~0.5 eV, correlating to a transfer of up to 0.5 e^−^, which aligns with a change in V_O_ concentration (Δδ ≈ 0.25) (*34*), and is further supported by our lattice parameter estimations (fig. S5). Moreover, the Co-*L* edge shift of ~0.8 eV between BM-SCO and PL-SCO implies an oxygen content of ~2.9 (SrCoO_2.9,_ or δ = 0.1) in the PL-SCO phase (*34*), reflecting a near-complete oxidation of the Co cations.

To rule out the possibility of electron beam irradiation–induced phase transitions, as reported in prior works (*32*, *35*, *36*), a control experiment without water vapor was performed under identical conditions (movie S2). No phase transition was observed even after more than 10 min of electron beam irradiation and heating. Moreover, oxidation did not occur even when a BM-SCO sample was heated at 200° to 300°C in an oxygen environment, even at a high O_2_ partial pressure of 0.2 mTorr (movie S3). In contrast, oxidation readily occurred at a water vapor pressure of ~0.01 mTorr, showing that BM-SCO is much more easily oxidized by water vapor than by O_2_. One possible explanation is that water molecules adsorb more readily on material surfaces than O_2_ (*37*). During the in situ ETEM experiment, water vapor adsorbs and dissociates into atomic hydrogen (H) and hydroxyl (–OH) species on the sample surface. Because the ionic radius of OH^−^ (95 pm) is smaller than that of O^2−^ (140 pm) (*37*), OH^−^ can more easily occupy the ordered oxygen vacancy sites than the incorporation of molecular O_2_. In addition, our DFT calculations confirm that OH^−^ has a lower insertion energy (Δ*E*_f_) than O^2−^, as shown in table S1, consistent with previous findings (*10*). This hydroxyl insertion initiates the oxidation of Co and triggers the structural transition toward higher oxidation states. Hydroxyl groups can also become mobile within the structure through proton hopping between oxide ions due to the low diffusion barrier of protons (*14*, *38*, *39*). Under electron beam irradiation and thermal heating in our in situ experiments, the incorporated hydrogen diffuses out of the film, leaving behind an oxidized SrCoO_*x*_ lattice. Recent studies have reported that protons derived from water dissociation can enhance oxidation by occupying interstitial sites in the oxide lattice (*14*). This mechanism lowers the V_O_ formation energy and reduces the diffusion barriers for both cations and anions, leading to more efficient oxidation in moist environments. This suggests that in this experiment, the protons behave as active atoms, facilitating the oxidation reactions, as shown in fig. S7.

To further investigate the electronic and structural changes as oxygen accumulates, we used DFT calculations (details in Materials and Methods). The band structure and density of states (DOS) for the G-type antiferromagnetic (G-AFM) ground state of BM-SCO and the newly formed IP-SCO phase are shown in fig. S8. The bandgap for BM-SCO is ~1.0 eV, indicating an insulating property, consistent with previous works (*40*). The ground state spin configuration of the ordered IP-SCO phase remains G-AFM, but the additional O atoms predominantly occupy states near the Fermi level, enhancing hybridization between Co d and O p orbitals. This enhanced hybridization leads to bandgap closure, imparting metallic behavior to the IP-SCO phase, similar to the IP-SrFeO_2.75_ phase (*4*).

### Structural evolution at the APB

**Fig. 3.**
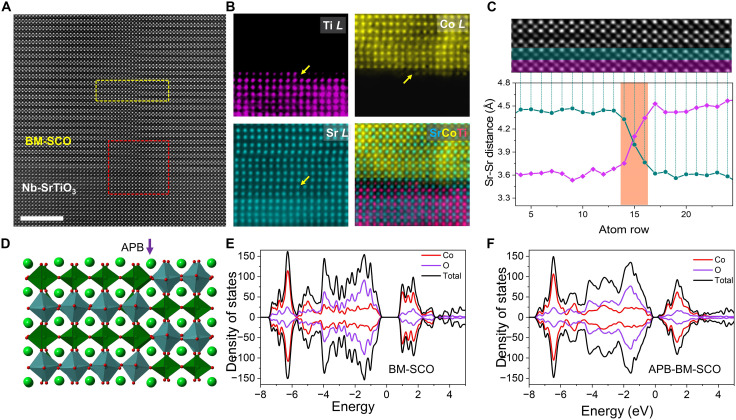
Atomic origin and structure of APBs in BM-SCO. (**A**) HAADF-STEM image displaying a typical APB where CoO_6_ and CoO_4_ sublayers are shifted by one half-cell. (**B**) Atomic-resolution EELS maps verifying the step terrace structure on the Nb-STO surface and the resulting APB. (**C**) HAADF-STEM image corresponding to the yellow dashed box area in (A) and out-of-plane Sr-Sr atomic distance variations across the APB, highlighting local lattice distortions. (**D**) Schematic structure model of the APB interface, showing the octahedral-to-tetrahedral sublayer transition. (**E** and **F**) Density of states (DOS) calculations comparing the APB region to a perfect crystal, revealing a bandgap reduction (from ~1 to ~0.3 eV) that could enable two-dimensional conduction pathways.

Figure 3A presents an HAADF-STEM image of a typical domain boundary in BM-SCO thin films. The CoO_6_ octahedra and CoO_4_ tetrahedra are shifted by one sublayer compared to adjacent domains, forming an APB. To understand the origin of this high-energy interface in BM-SCO thin films, the intersection of domain boundaries with the film/substrate interface was evaluated using spherical aberration–corrected HAADF-STEM and atomic-resolution EELS maps, as shown in Fig. 3 (A and B). These results reveal that the BM-SCO thin-film growth begins with an octahedral sublayer at the film/substrate interface (*41*), consistent with our DFT calculations (fig. S9). As a result, APBs in BM-SCO form at the STO substrate terrace steps. Because the half-cell of BM-SCO shifts out-of-plane at each step terrace, an APB arises where an octahedral layer on one side connects to a tetrahedral layer on the other side, as shown in Fig. 3 (C and D). A variation in interplanar distance is evident when crossing the APB (Fig. 3C).

DFT calculations were further used to investigate the stability of these APBs. An APB interface model was constructed by shifting one half-crystal along [001] by one sublayer spacing relative to the other, as depicted in Fig. 3D. As noted, when the tetrahedral layer crosses the APB, it transforms into an octahedral layer, creating substantial strain at the boundary due to the large lattice distance mismatch, as displayed in Fig. 3 (C and D). To understand the effect of the APB on its electronic structure, the total electronic DOS was compared with that of the perfect crystal. As shown in Fig. 3 (E and F), a shift of the conduction band states toward the Fermi level appears, reducing the bandgap from ~1 to ~0.3 eV. This suggests that in the insulating BM-SCO, the closure of the bandgap at APBs may enable the formation of two-dimensional conductive channels.

**Fig. 4.**
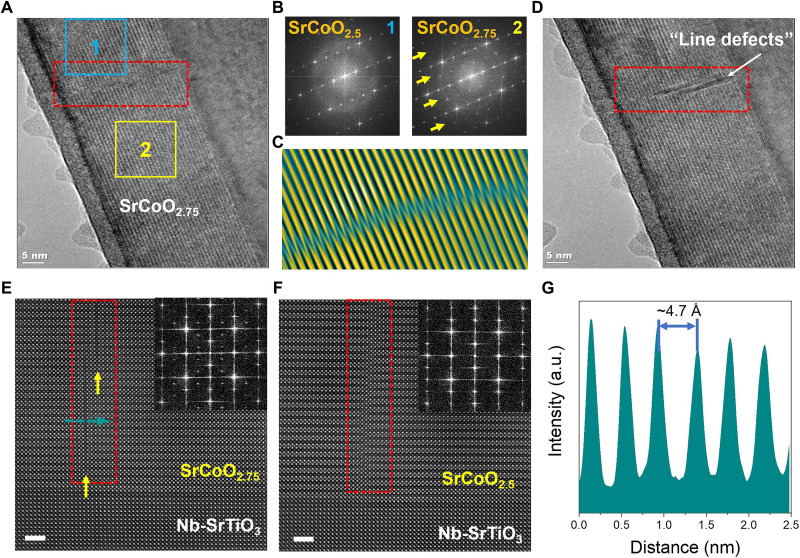
Dynamic evolution of APBs under water vapor oxidation. (**A**) A high-resolution TEM image capturing the reaction front at an APB. (**B**) FFT patterns associated with the BM-SCO and IP-SCO regions marked in (A). (**C**) Inverse FFT corresponding to the red box area in (A). (**D**) High-resolution TEM image after the reaction interface passes the APB, demonstrating the formation of a defect structure. (**E** and **F**) HAADF-STEM image showing the reversible transformation between the generated defect structure, a nanoscale gap, and APB at the atomic scale during oxidation and reduction, respectively. Scale bars, 2 nm. (**G**) Line intensity profile corresponding to the cyan dashed line in (E) showing the Sr-Sr interatomic distance (~4.7 Å) across the formed defect.

The structural evolution at the APB during in situ water vapor oxidation was further investigated. When the phase transition direction is parallel to the boundary, as shown in movie S4, the APB minimally affects the oxidation process, with the only observable consequence being the formation of “line defects” along the boundary after oxidation. In contrast, if the oxidation front moves perpendicular to the APB, significant strain at the boundary causes a temporary blockage, highlighting the APB’s structural impact. As shown in Fig. 4 (A to C), the reaction front is sharply defined and remains stationary at the APB, with the BM-SCO structure preserved above the boundary, while below it transforms into the IP-SCO phase, as confirmed by the FFT patterns in Fig. 4B. The considerable strain present at the APB obstructs the phase transition across this boundary, consequently influencing the transition speed along this direction. Conversely, the phase transition occurring parallel to the APB remains unaffected by this phenomenon. Similar effects have been documented in other studies (*42*–*44*). Upon crossing the APB, the oxidation front leaves two distinct line defects, likely due to lattice contraction during the oxidation, as illustrated in the red dashed box in Fig. 4D. Figure 4E further shows the formation of these line defects or small gaps in STEM mode, where the Sr-Sr interatomic distance measures ~4.7 Å (Fig. 4G), notably larger than the typical tetrahedral layer spacing. This is because the in-plane Sr-Sr distance in IP-SCO (~3.8 Å) is slightly shorter than that in BM-SCO (~3.9 Å). This difference results in the stretching of the interatomic distance at the APB, owing to the irregular and weaker bonding characteristics of this interface, ultimately leading to the formation of line defects. This suggests that the APB can effectively accommodate the strain from IP-SCO lattice contraction. The gap structure can transform back into the APB during reduction from IP-SCO to BM-SCO under electron beam irradiation (Fig. 4F). This reversibility indicates that gap formation does not cause structural collapse but rather preserves the underlying chemical bonds. This insight underscores that the role of APBs is not just to accommodate strain but also to provide flexibility for reversible phase transformations, an attribute that could be crucial for tuning the material’s functional properties.

## DISCUSSION

In summary, our in situ ETEM experiments demonstrate that BM-SCO undergoes a water vapor–driven, multistep transition to IP-SCO and lastly to PL-SCO. Our findings also demonstrate that the APBs within BM-SCO, which originate from the step terraces of the substrates, play crucial roles in the reversible phase transition. As the reaction progresses through the APBs, a reversible substantial structural distortion is observed, resulting in strain relaxation due to lattice contraction during the oxidation process. Our atomic-scale observations clarify the water vapor–driven TPT mechanism and the enhanced mass transport facilitated by water vapor in vacancy-ordered oxides. These insights not only elucidate oxidation processes in BM-SCO but also pave the way for understanding analogous phenomena in vacancy-ordered materials such as SrFeO_2.5_ (*3*, *45*), CaFeO_2.5_ (*41*, *46*), La_1−*x*_Sr*_x_*MnO_3_ (*47*, *48*), and SrCrO_2.8_ (*49*, *50*) that exhibit similar structural features.

## MATERIALS AND METHODS

### Thin-film growth

SrCoO_2.5_ thin films (~30 nm thick) were grown on (001)-oriented STO substrates using a customized pulsed laser deposition system equipped with reflection high-energy electron diffraction. A krypton fluoride excimer laser (λ = 248 nm) was operated at a fluence of 1.2 J cm^−2^ and a repetition rate of 2 Hz. The substrate temperature was maintained at 750°C in an oxygen partial pressure of 100 mTorr during growth.

### STEM and ETEM

A focused ion beam (Helios) was used to prepare cross-sectional samples for STEM and in situ TEM. The sample surfaces were coated with Pt and C to protect them from Ga ion damage. At an accelerating voltage of 30 kV and a current of 0.46 nA, the cross-sectional lamella was reduced to ~200 nm thickness, followed by a fine polish at 5 and 2 kV and a low current of 80 pA. The final thickness of the lamella was ~50 nm.

HAADF-STEM, ABF-STEM, and EELS mapping were conducted on a JEM ARM200F equipped with a probe aberration corrector. For HAADF and ABF imaging, the collection angles were 90 to 370 and 10 to 23 mrad, respectively, using a probe current of ~23 pA and a pixel time of 16 μs. EELS mapping and valence analysis were carried out with a 0.25-eV dual collection and channel dispersion with a pixel time of 0.01 s.

An FEI Titan ETEM, equipped with an objective-lens aberration corrector, was used to conduct in situ TEM investigations. A Gatan furnace-based heating holder enabled observations under controlled temperature and water vapor pressure. The SCO lamellae were exposed to ambient air during transfers between ETEM and STEM. Each transfer involved air exposure—typically on the order of several hours—but in some cases, samples were stored in ambient conditions for up to several days. The lamellae remain structurally and chemically stable in air over these timescales.

### DFT calculation

These calculations were performed using the Vienna ab initio simulation package (VASP) (*51*, *52*) and the exchange-correlation functional by Perdew–Burke–Ernzerhof modified for solids within generalized gradient approximation (GGA) (*52*). Projector-augmented wave potentials provided as a part of the VASP package were used to approximate the effect of the core electrons (*53*) in the atomic electronic configurations: [Ar 3d^10^]4s^2^4p^6^5s^2^ for Sr; [Ne 3s^2^]3p^6^3d^3^4s^1^ for Ti, [Ne 3s^2^]3p^6^3d^7^4s^1^ for Co, and [He]2s^2^2p^4^ for O. The calculations were performed for the 3 × 3 × 1 and 4 × 4 × 1 k mesh, which yielded nearly identical results. The plane-wave basis-set cutoff was set to 500 eV. The total energy and force convergence criteria were set to 10^−5^ eV and 0.01 eV/Å, respectively. A *k* mesh of 2 × 4 × 1 was adopted for an APB supercell (consisting of 216 atoms). We introduced the on-site Coulomb interaction of *U*_eff_ = 4.0 eV by means of the GGA + U scheme (*54*), where the *U*_eff_ value has been successfully used in the previous report (*16*).
