# Supplementary material for: In situ atomic-resolution imaging of water vapor–driven multistep oxidation dynamics in strontium cobaltite
Source: Sci Adv. 2025 Aug 22;11(34):eadx8890. doi: 10.1126/sciadv.adx8890 (PMC12372878; doi:10.1126/sciadv.adx8890)
Supplement: Supplementary file 1 — Figs. S1 to S9 Supplementary Text Table S1 Legends for movies S1 to S5 [file sciadv.adx8890_sm.pdf]

Science Advances
AAAS

Supplementary Materials for

In situ atomic-resolution imaging of water vapor–driven multistep oxidation dynamics in strontium cobaltite

Zhenzhong Yang *et al.*

Corresponding author: Zhenzhong Yang, zzyang@phy.ecnu.edu.cn; Yingge Du, yingge.du@pnnl.gov

Sci. Adv. **11**, eadx8890 (2025)
DOI: 10.1126/sciadv.adx8890

The PDF file includes:

Figs. S1 to S9
Supplementary Text
Table S1
Legends for movies S1 to S5

Other Supplementary Material for this manuscript includes the following:

Movies S1 to S5

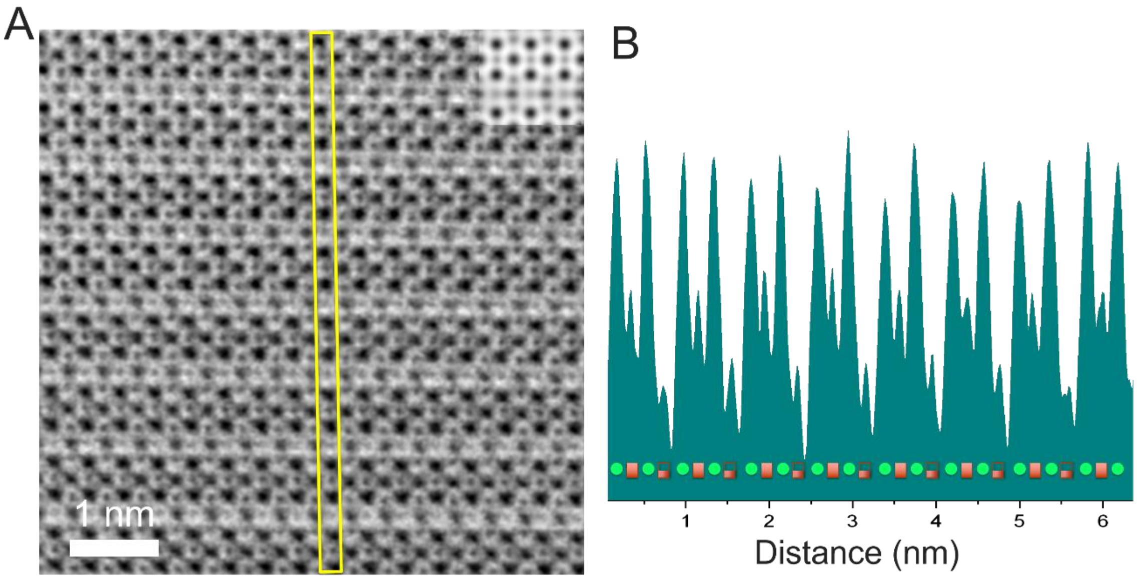

Fig. S1 The oxygen vacancy channels in BM-$SrCoO_{2.5}$. (**A**) ABF STEM image of $SrCoO_{2.5}$ viewed along [100] zone axis, with the simulated image overlaid. (**B**) Line intensity profile corresponding to the yellow rectangular box area in (**A**). The full-filled and half-filled brown squares are used to indicate the oxygen occupation. The Sr columns are indicated using green spheres.

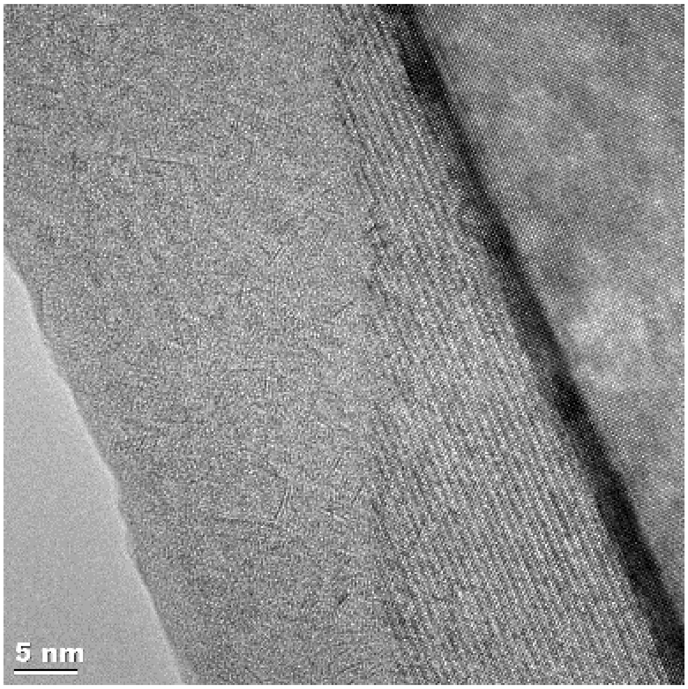

Fig. S2 The HRTEM image shows the formation of an amorphous phase in SCO thin film after exposure to water vapor at room temperature.

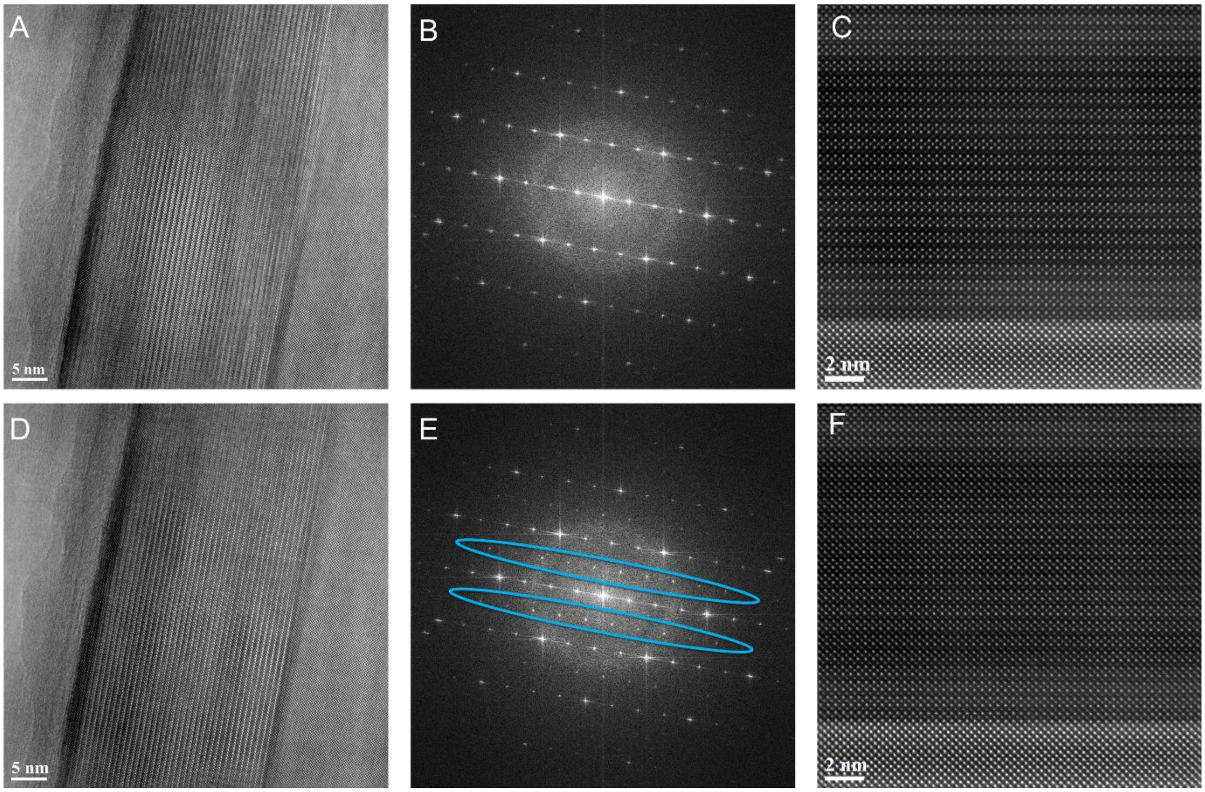

Fig. S3 HRTEM images showing the atomic structure change between $SrCoO_{2.5}$ and $SrCoO_{2.75}$. (**A, D**) HRTEM images of $SrCoO_{2.5}$ and $SrCoO_{2.75}$, respectively. (**B**, **E**) FFT pattern corresponding to (**A**) and (**D**), respectively. (**C**, **F**) HAADF-STEM of $SrCoO_{2.5}$ and $SrCoO_{2.75}$, respectively.

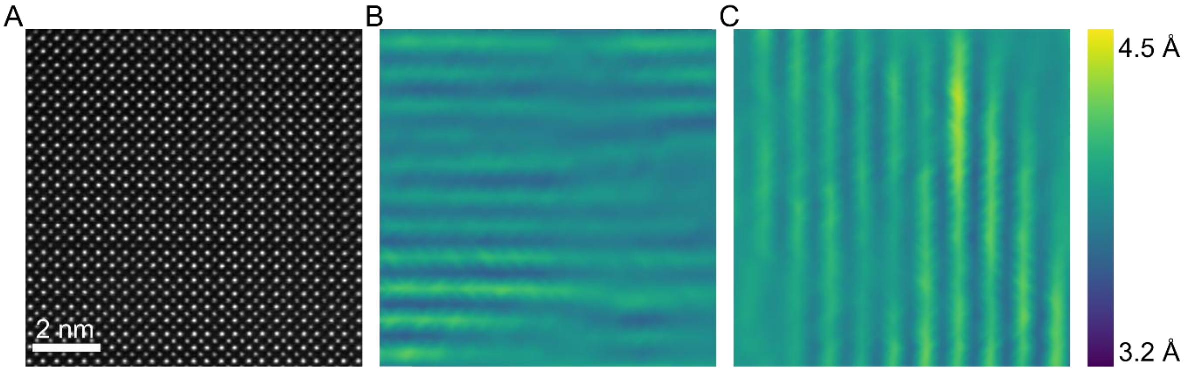

Fig. S4. **The HAADF image shows the transition process from IP-SCO to PL-SCO.** (A) HAADF-STEM image and corresponding (B) out-of-plane and (C) in-plane lattice distance maps.

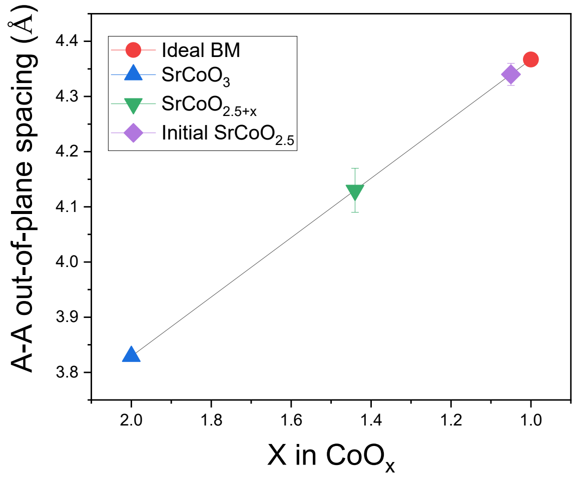

Fig. S5 Determination of oxygen content in SCO. Lattice expansivity as a function of oxygen deficiency (x in CoO_x).

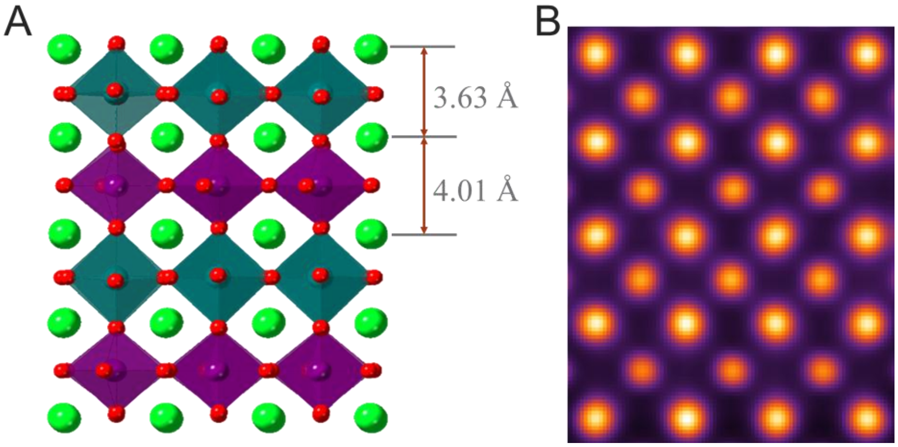

Fig. S6 The crystal structure of $SrCoO_{2.75}$. (**A**) DFT calculated $SrCoO_{2.75}$ crystal structure and (**B**) corresponding simulated HAADF-STEM image.

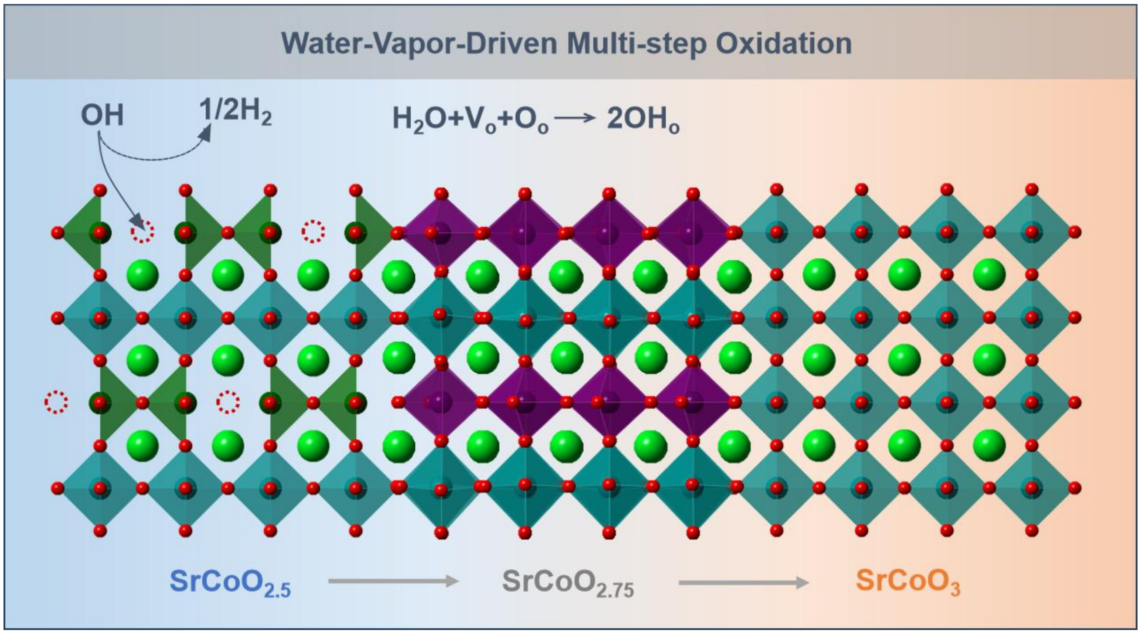

Fig. S7 The schematic diagram of the phase transition from BM-$SrCoO_{2.5}$ to $SrCoO_3$.

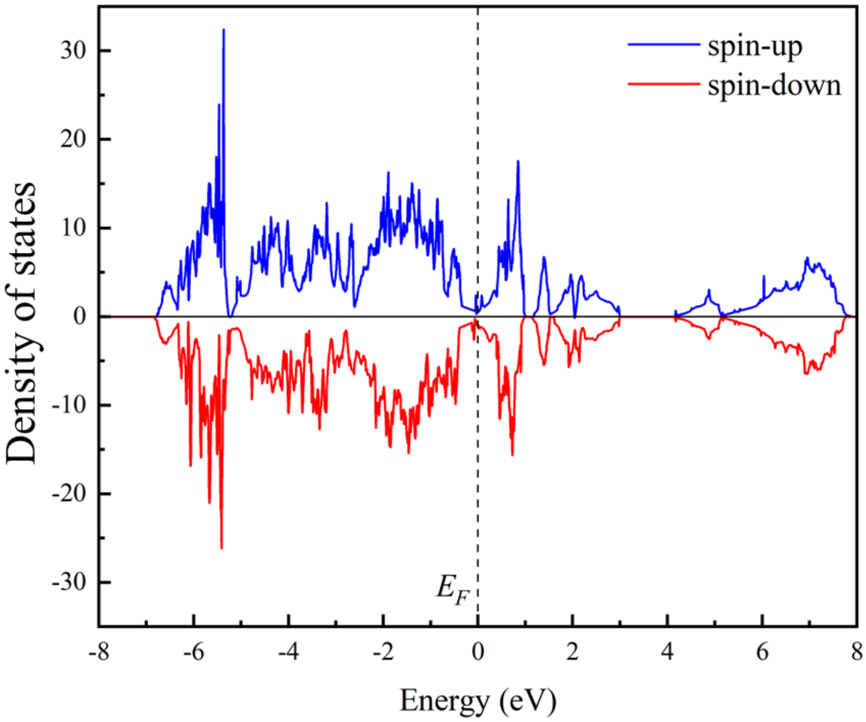

Fig. S8 Density of states of IP-SCO.

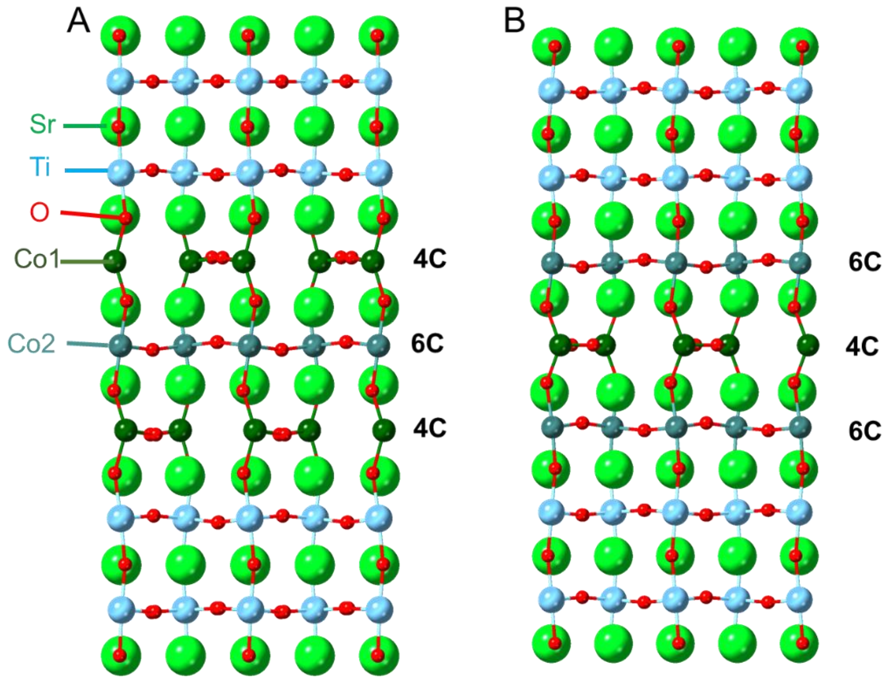

Fig. S9. Ball-and-stick representations of the SCO/STO heterostructures used to calculate the interfacial energy difference for SCO. Heterostructure models terminated with CoOx planes containing 4-coordinated (4C) and 6-coordinated (6C) units of CoO_4 (**A**) and CoO_6 (**B**), respectively.

To evaluate relative stability of the $SrCoO_{2.5}$/STO (001) interfaces terminated with CoO_x planes containing CoO_4 tetrahedra (4C) and CoO_6 octahedra (6C), we used the periodic heterostructure models shown in Fig.S6. For simplicity, these two models are referred to as 4C-4C and 6C-6C, respectively. In both cases, the lateral cell corresponds to the √2×√2 crystallographic cell of the STO (001) surface. Both systems contain four TiO_2 and three CoO_x planes alternated with SrO planes. By construction, these systems are symmetric relative to the central CoO_x plane and their compositions ($Sr_{14}Co_6Ti_8O_{38}$ and $Sr_{14}Co_6Ti_8O_{40}$) differ by two oxygen atoms only. Accordingly, we took the difference of the total energies, corrected for the energy of a gas-phase O_2 molecule and normalized for the interface area as the measure of their relative stability.

While the bulk SCO was reported to have the G-type antiferromagnetic structure, the interfacial interactions may affect the spin configuration in the ultra-thin SCO layers. Therefore, we first calculated the relative energies in the non-spin-polarized approximation. By optimizing both the lattice parameters and internal coordinates of all atoms and accounting for the energy of the O_2 molecule, we arrived at the energy difference between the 4C-4C and 6C-6C structures of ~4.7 eV. Normalizing this energy for the interface area, we arrived to the 6C interface being more stable than the 4C interface by 77 meV/Å^2 or ~1.17 eV per interfacial Co atom.

To access the possible effect of the interface with STO on the magnetic ordering in SCO, we calculated the energies of $SrCoO_{2.5}$ in G-, A-, and C-type antiferromagnetic configurations and ferromagnetic (FM) configuration. These calculations were performed for the lattice parameters determined experimentally, while the internal coordinates of atoms were fully optimized. The total energies of these configurations vary by ~1 eV (from A-type to be most stable and FM being least stable), i.e., ~125 meV per Co atoms. Thus, the difference in the interfacial energies between 4C and 6C interfaces approximately an order of magnitude larger than the possible effects of the magnetic ordering. In other words, the details of the spin arrangements at the interface do not affect the thermodynamic preference for the 6C / STO interface.

Finally, we note that the lateral parameters of the fully optimized 4C-4C and 6C-6C supercell are 0.2–0.5% smaller than those of pure STO, i.e., the SCO layer is subjected to lower tensile strain than in practice. To more accurately account for the effect of the STO-induced epitaxial strain, the lateral parameters of both 4C-4C and 6C-6C models were fixed at the values corresponding to the STO bulk (√2×3.905 Å) and the total energies of these systems were minimized with respect to

the out-of-plane supercell parameter. We found that this correction has negligible effect in both spin-polarized and non-spin-polarized energies.

	SrCoO2.5	SrCoO2.75	SrCoO2.75H
E_{AFM}	-113.85716 eV	-118.77998 eV	-123.43109 eV
E_{FM}	-113.20417 eV	-118.49512 eV	-122.97251 eV
ΔE	-0.65299 eV	-0.28486 eV	-0.45858 eV
$E_{SrCoO_{2.5}}$		-112.29878 eV	-113.21091 eV
$E_{(O)^{2-}}$		-7.9494596 eV	
$E_{(OH)^{-}}$			-7.1372899 eV
ΔE_f		1.4682596 eV	-3.0828901 eV

Table 1

$SrCoO_{2.75}H$ refers to the system in which two -OH groups are inserted to the super-cell used to form the $H_2(SrCoO_{2.75})_8$ phase. The formation energies ΔE_f of $SrCoO_{2.75}$ and $SrCoO_{2.75}H$ are calculated as follows:

$$\Delta E_f = E_{SrCoO_{2.75}} - E_{SrCoO_{2.5}} - E_{(O)^{2-}} \quad (S1)$$

$$\Delta E_f = E_{SrCoO_{2.75}H} - E_{SrCoO_{2.5}} - E_{(OH)^{-}} \quad (S2)$$

Here, $E_{SrCoO_{2.5}}$ represents the isolated energy of $SrCoO_{2.5}$. The terms $E_{(O)^{2-}}$ and $E_{(OH)^{-}}$ represent the total energies of isolated O^{2-} and OH^{-} ions, respectively.

Movie S1.

In-situ atomic-scale observation of dynamical phase transition processes in BM-SCO thin film under ~0.01 mTorr water vapor at 100 °C. The video is accelerated by the factor of 20 relative to the actual time.

Movie S2.

In-situ atomic-scale observation of the BM-SCO/STO under 200 °C heating without water vapor. The video is accelerated by the factor of 40 relative to the actual time.

Movie S3.

In-situ atomic-scale observation of the BM-SCO/STO under ~0.2 mTorr O_2 at 250 °C. The video is accelerated by the factor of 10 relative to the actual time.

Movie S4-S5.

In-situ observation of dynamical phase transitions in the APBs areas in BM-SCO thin film under ~0.2 mTorr water vapor at 250 °C. The video is accelerated by the factor of 5 relative to the actual time.
